# Two-Year Clinical Outcome of Using Biphasic Osteochondral Constructs in the Treatment of Patients with Mild-to-Moderate-Stage Osteoarthritis of the Knee

**DOI:** 10.3390/bioengineering13070778

**Published:** 2026-07-04

**Authors:** Chih-Yung Chiang, Po-Wei Lee, Chang-Chin Wu

**Affiliations:** 1 Department of Biomedical Engineering, National Taiwan University, Taipei 106216, Taiwan; okay0506@gmail.com; 2 Department of Orthopedic Surgery, College of Medicine, National Taiwan University, Taipei 100233, Taiwan; 3 BioGend Therapeutics Co., Ltd., Taipei 115603, Taiwan; powei.lee@biogend.com.tw

**Keywords:** knee osteoarthritis, biphasic osteochondral construct, single-stage autologous cartilage implantation, cartilage repair, clinical feasibility

## Abstract

A biphasic osteochondral construct has been developed to repair focal chondral and osteochondral lesions. This study aimed to evaluate the safety and preliminary clinical feasibility of using the biphasic osteochondral construct in treating patients having mild-to-moderate-stage knee osteoarthritis. This single-center, prospective, open-label, single-arm feasibility trial compared 2-year knee functional outcome (KOOS and IKDC scores), pain VAS score, and radiological assessment of repaired cartilage (MOCART score) after treatment versus the corresponding preoperative scores. All enrolled patients had a clinical history of primary knee osteoarthritis (OA), Kellgren Lawrence grade 1–3 without valgus or varus deformity greater than 5 degrees. Five men and three women completed the two-year follow-up, with a mean age of 53.9 ± 9.3 years (range 34–63 years). The average lesion size was 4.5 ± 2.9 cm^2^ (range 2–9 cm^2^). The patients received one to three biphasic osteochondral constructs. No major adverse effects or complications were reported postoperatively. The mean KOOS subscale values, VAS scores, and IKDC scores were all improved significantly at six months post operation and were maintained for two years. The changes in MOCART scores of the regenerated cartilage were parallel to the changes observed in KOOS values. Our findings provide preliminary evidence of the safety and clinical feasibility of using biphasic osteochondral constructs to treat focal chondral and osteochondral lesions in patients with mild- or moderate-stage osteoarthritis, and support the rationale for larger controlled trials.

## 1. Introduction

Articular cartilage lesions of the knee are common, affecting approximately 60% of patients undergoing knee arthroscopy, with symptomatic chondral defects reported in up to 36% of the general population [1,2]. Focal chondral and osteochondral lesions arising from trauma or degenerative pathology carry significant clinical burden, as the limited regenerative capacity of articular cartilage predisposes affected joints to the gradual onset of osteoarthritis (OA), resulting in debilitating joint pain and impaired quality of life [3,4]. Without effective interventions to delay or prevent joint deterioration, OA poses a significant clinical challenge for orthopedic surgeons [4]. Surgical options are limited and vary depending on factors such as lesion area and depth, disease stage, patient age, and pathologies involving the subchondral bone and other joint structures [5]. Furthermore, current treatments typically do not provide a long-term benefit [6]. Consequently, total joint replacement remains the only treatment option for symptomatic relief and functional restoration in patients with end-stage OA.

To repair cartilage lesions, numerous surgical approaches, such as microfracture, osteochondral autograft transplant (mosaicplasty), and autologous chondrocyte implantation (ACI), have been proposed [4]. While bone marrow mesenchymal stem cells with chondrogenic differentiation potential can be introduced into the defect site during microfracture surgery, the resultant formation of fibrocartilage is mechanically inferior to the hyaline cartilage in the normal articular surface [7,8]. Improvements in clinical outcomes are observed in the early postoperative period; however, an accelerated deterioration with a high failure rate during the following two years has been reported [4,9]. Moreover, it has been reported that microfractures may lead to a negative effect on subsequent treatments in the event of failure [10,11].

For mosaicplasty, osteochondral grafts collected from non-weight-bearing sites of the joints are placed at the articular lesion [12]. Mosaicplasty, through the delivery of autologous chondrocytes with native hyaline matrix, has been demonstrated to have outstanding clinical results [13]. However, the procedures are technically demanding; thus, problems frequently arise during surgery. Moreover, the harvest of a large amount of osteochondral tissue has been reported to be associated with a higher rate of donor site morbidity [13].

As a cell-based therapeutic approach, ACI involves autologous chondrocyte collection from a low-weight-bearing site, in vitro expansion, and cell transplantation into the defect site [14]. Although positive clinical outcomes have been confirmed, multiple operations and prolonged recovery periods are needed. Furthermore, technical challenges such as leakage of the engrafted cells, inhomogeneous cell distribution, and periosteal flap hypertrophy remain major clinical concerns [15]. To better secure the implanted cells, matrix-induced ACI (MACI) has been developed, in which autologous chondrocytes are cultivated on biomaterial-based scaffolds before implantation. However, whether MACI is superior to other treatment approaches in terms of long-term clinical outcomes is controversial [16,17].

Compared with chondrocytes that are isolated from native tissue and cultivated in vitro, minced cartilage tissues that are inherent to the extracellular matrix can much more precisely mimic the natural microenvironment of articular cartilage, thereby effectively supporting chondrocyte phenotype maintenance [18,19]. Furthermore, minced articular cartilage can be prepared with implantation immediately after graft harvest [20]. Without a prolonged cell expansion stage and second operation, minced cartilage tissues have been considered an attractive cell source for MACI.

Chiang et al. reported the safety and effectiveness of using a biphasic osteochondral construct for autologous cartilage implantation in a two-year follow-up [21]. Furthermore, five-year outcomes revealed significant improvements in the mean Knee Injury and Osteoarthritis Outcome Score (KOOS), magnetic resonance observation of cartilage repair tissue (MOCART) score, and computed tomography and histological findings [22]. Through the implantation of minced articular cartilage using biphasic osteochondral constructs, the osteochondral lesion was covered with hyaline-like cartilage that remained durable for a minimum of five years [22]. Although the safety and effectiveness of using biphasic osteochondral constructs for treating chondral and osteochondral lesions have been verified, patients with diffuse degenerative changes such as OA in the knee were not fully evaluated in those trials reported. It has been well established that osteochondral defects present an essential risk factor for the onset of secondary OA [23], while primary OA can lead to cartilage lesions [24]. We, therefore, aimed to determine the safety and preliminary clinical feasibility of using a biphasic osteochondral construct for autologous cartilage implantation in an extended patient population with mild- to moderate-stage osteoarthritis in the knee. An open-label and single-arm trial was performed to evaluate two-year outcomes after treatment of patients with OA knee. The International Knee Documentation Committee (IKDC)-2000 subjective knee evaluation score, KOOS, visual analog scale (VAS) score, MOCART score, and cartilage thickness were assessed to evaluate the outcomes.

## 2. Methods

### 2.1. Patients

A single-center, investigator-initiated, prospective, open-label, single-arm trial was conducted from January 2019 to December 2022. The study protocol was reviewed and approved by the Institutional Review Board of En Chu Kong Hospital, New Taipei City, Taiwan (approval no. ECKIRB1080501), and the Ministry of Health and Welfare, Taiwan (approval no. 1086800526). This trial was registered at ClinicalTrials.gov with the identifier NCT05924763. The trial complied with the national legal regulations and the tenets of the Helsinki Declaration. Informed consent was obtained from all patients prior to inclusion. Safety was defined as the absence of major adverse events including infection, implant failure, or mechanical collapse of the load-bearing surface, and the resolution of any minor adverse effects (e.g., swelling, soreness) by three months postoperatively. The clinical feasibility of RevoCart was assessed by evaluating whether statistically significant improvements from baseline were observed in KOOS subscale scores, IKDC scores, and VAS pain scores at 24 months postoperatively. The MOCART score was used as an imaging biomarker to assess the radiological quality of the repaired cartilage.

Consecutive patients who met the following inclusion criteria were invited to participate in this trial: (1) age of 16–65 years, (2) symptomatic chondral or osteochondral lesions of the knee, which may include but were not limited to multiple lesions or lesions that had failed microfracture or mosaicplasty, (3) lesion requiring primary surgical intervention, (4) the index lesion classified as an International Cartilage Repair Society grade 3–4 lesion with Kellgren–Lawrence (KL) grade 1–3, Outerbridge grade 4, or Osteochondritis dissecans grade 3–4, and (5) willingness and ability to consent to participate in the study.

The exclusion criteria were as follows: (1) rheumatoid arthritis or other inflammatory arthritis, (2) index cartilage lesion requiring four or more implants, (3) skeletal immaturity, defined as unclosed epiphyses based on plain roentgenography, (4) concomitant comorbidities, such as anterior cruciate ligament instability, significant knee instability, or significant malalignment that would not be corrected before or during the study procedure, (5) local or systemic infection, and (6) pregnancy or breastfeeding.

### 2.2. Surgical Procedure

The surgical procedure was conducted following the manufacturer’s manual as described previously [21,22]. Briefly, arthroscopic examination and debridement were performed to confirm the location and size of the lesions and estimate the number of implants to be applied. The number of biphasic constructs used (one to three) was determined by the total area of the chondral or osteochondral lesion, assessed intraoperatively. Each implant covers approximately 1.4 cm^2^ of lesion area and was press-fitted into a prepared cylindrical recipient site, with a 2–3 mm interval maintained between adjacent constructs. In patients with larger lesions, the biphasic constructs were prioritized for the weight-bearing portion of the defect, while any residual non- or less-weight-bearing area received supplementary marrow stimulation (microfracture or microdrilling) to achieve complete lesion coverage.

**Figure 1:**
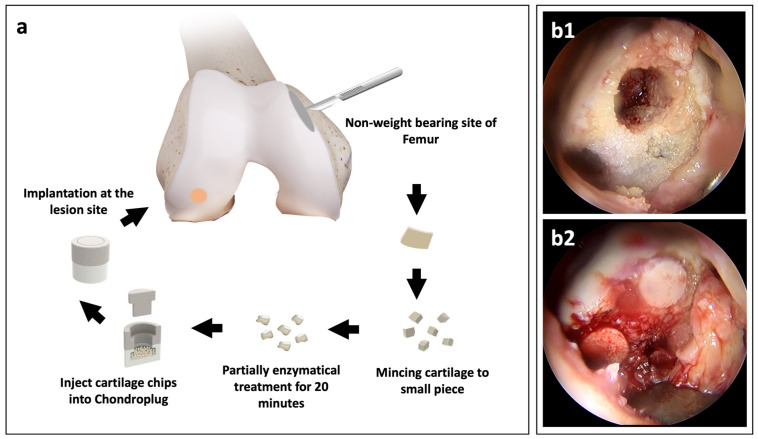
Implantation of cartilage-loaded construct prepared using the one-step autologous cartilage repair system (RevoCart) into the osteochondral lesion site. (**a**) Illustration of RevoCart surgical procedure. (**b1**) Recipient lesion was drilled into a cylindrical pit. (**b2**) The prepared biphasic construct was pressed into the recipient site.

For each implant, approximately 100 mg of cartilage tissue was collected from a non-weight-bearing area of the femur (such as lateral or medial superior trochlea ridge or lateral intercondylar notch). The tissue was immersed in a normal sterile solution and mechanically minced in a dedicated cartilage processor (BioGend Therapeutics, Taipei, Taiwan) for 2 min into fine particles less than 1 mm in diameter. Next, these particles were further enzymatically treated with collagenase blend solution (RevoCart Enzyme, BioGend Therapeutics, Taipei, Taiwan) at 37 °C for 20 min, followed by multiple cycles of saline washes to remove any residual enzmym. Then, the processed cartilage particles were collected and transferred into a biphasic porous scaffold (Chondroplug, BioGend Therapeutics, Taipei, Taiwan). The recipient hole was prepared drilling pits into the subchondral cancellous bone with a diameter and depth of 8.5 mm. Finally, the graft-laden scaffold was pressed into the pits. The surgical procedure is illustrated in Figure 1a, and scaffold implantation through arthroscopy is demonstrated in Figure 1(b1,b2).

For the first six weeks after operation, the knee range of motion was restricted between 0° and 90° of flexion using a knee brace, and only partial weight bearing (weight of leg) on the operated limb was allowed. Continuous passive motion devices were used four times daily, with 100 cycles each session. After six weeks, the operated leg was allowed to bear weight as tolerated.

### 2.3. Follow-Up Assessment

The IKDC, KOOS, and VAS scores were evaluated preoperatively and at 6 weeks and 3, 6, 12, and 24 months postoperatively. To assess the quality of the regenerated cartilage tissue, plain radiographs and MRI were conducted preoperatively and at one and two years post operation. An independent radiologist reviewed and analyzed the MRI images according to the revised MOCART scale.

### 2.4. Statistical Analysis

The values obtained at each follow-up were compared with the preoperative scores using the Wilcoxon signed rank test for mean KOOS values and mean VAS scores. The subscales of the mean KOOS values at various time points were compared using the Kruskal-Wallis test with Dunn’s test as the post hoc analysis. The MOCART scores of the regenerated cartilage at different postoperative stages were compared using Cochran’s Q test with McNemar’s test as the post hoc test. Statistical analyses were conducted with SPSS software (version 20.0.0, SPSS Inc., Chicago, IL, USA). All results are presented as the mean ± standard deviation with 95% confidence intervals (95% CI). For each comparison, differences with *p* < 0.05 were statistically significant. Non-parametric analyses were selected because the sample size (*n* = 8) was insufficient to assume normality. Retrospective power analysis based on the observed IKDC improvement (mean difference: 42 points; SD: 14 points) indicated approximately 95% power at α = 0.05 using the Wilcoxon signed-rank test. No correction for multiple comparisons was applied, given the exploratory and hypothesis-generating nature of this feasibility study. Effect sizes were estimated using Cohen’s d for paired comparisons. A Spearman correlation analysis was performed between the change in total MOCART score and change in IKDC score at 24 months.

## 3. Results

Of the nine initially screened and enrolled patients, one failed to complete the formal follow-up program due to subsequent withdrawal from the clinical trial. The remaining eight patients successfully completed the full 2-year postoperative longitudinal surveillance protocol, and their detailed individualized baseline demographic data are outlined in Table 1. This evaluable cohort consisted of five men and three women with a mean age of 53.9 ± 9.3 years (range: 34–63 years). All included subjects had a confirmed clinical diagnosis of symptomatic, primary knee OA, staged at KL grades 1 to 3, presenting without valgus or varus deformities exceeding 5 degrees. The average lesion size was 4.5 ± 2.9 cm^2^, spanning a wide clinical spectrum from 2 to 9 cm^2^. Three patients received one RevoCart implant, four patients received two implants, and two patients received three implants. No major adverse effects or complications were reported postoperatively.

To achieve customized anatomical reconstruction based on intraoperative defect geometry, three patients received a single RevoCart implant, four received two, and two required three constructs. Additionally, to manage the complex, multi-tissue degenerative nature of authentic OA, concomitant procedures were performed alongside the biphasic construct placement where indicated; specifically, three patients underwent microdrilling and one received synchronous meniscal repair. Notably, no major treatment-related adverse events or complications were reported postoperatively, demonstrating an encouraging initial safety profile within this clinically representative cohort.

**Figure 2:**
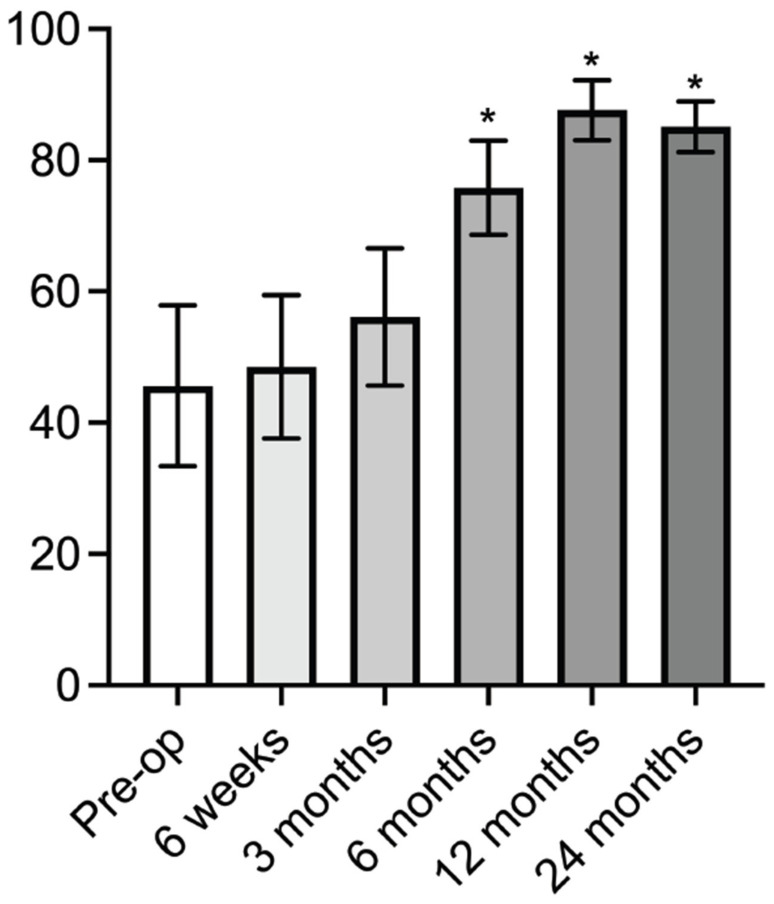
The International Knee Documentation Committee (IKDC) subjective score at preoperation and at 6 weeks and 6, 12, and 24 months post operation. Error bars indicate mean ± SD; *n* = 8. * Significant improvement when compared to the Pre-op value.

**Figure 3:**
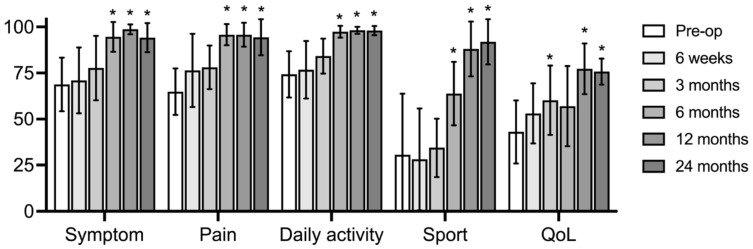
The Knee Injury and Osteoarthritis Outcome Score (KOOS) at preoperation and at 6 weeks and 6, 12, and 24 months post operation. QoL, quality of life. Error bars indicate mean ± SD; *n* = 8. * Significant improvement when compared to the Pre-op value.

**Figure 4:**
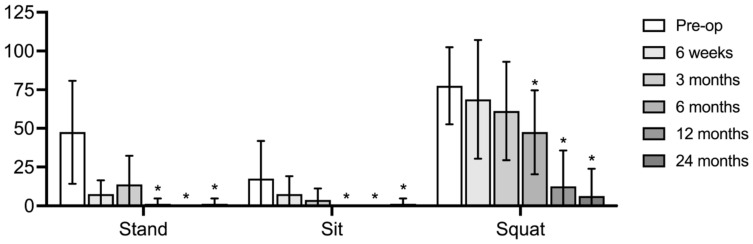
The visual analog scale (VAS) score for pain at preoperation and at 6 weeks and 6, 12, and 24 months post operation. Error bars indicate mean ± SD; *n* = 8. * Significant improvement when compared to the Pre-op value.

Figure 2 shows the IKDC scores before surgery and at 6 weeks, as well as 3, 6, 12, and 24 months after surgery. The preoperative mean IKDC score was 45.6 ± 12.3 (95% CI: 35.3–55.9), significantly increasing to 75.8 ± 7.2 (95% CI: 69.8–81.8) at six months postoperatively (*p* = 0.0078; Cohen’s d = 2.73; Figure 2). Similar improvements were observed in the KOOS subscales of symptom (68.8 ± 14.5 vs. 94.2 ± 7.9; *p* = 0.0078; d = 2.17), pain (64.9 ± 12.6 vs. 94.4 ± 9.7; *p* = 0.0078; d = 2.68), daily activity (74.3 ± 12.6 vs. 98.0 ± 2.5; *p* = 0.0078; d = 2.62), and sport (30.6 ± 33.2 vs. 91.9 ± 12.2; *p* = 0.0156; d = 2.40) at 24 months postoperatively compared to preoperative scores (Figure 3). During postoperative follow-up, there was a significant decrease in mean VAS scores for Standing (47.5 ± 33.3 vs. 1.3 ± 3.5; *p* = 0.0078), Sitting (17.5 ± 24.3 vs. 1.3 ± 3.4; *p* = 0.0078), and Squatting (77.5 ± 24.9 vs. 6.3 ± 17.7; *p* = 0.0078), comparing preoperative scores to those from 24 months post operation (Figure 4).

**Figure 5:**
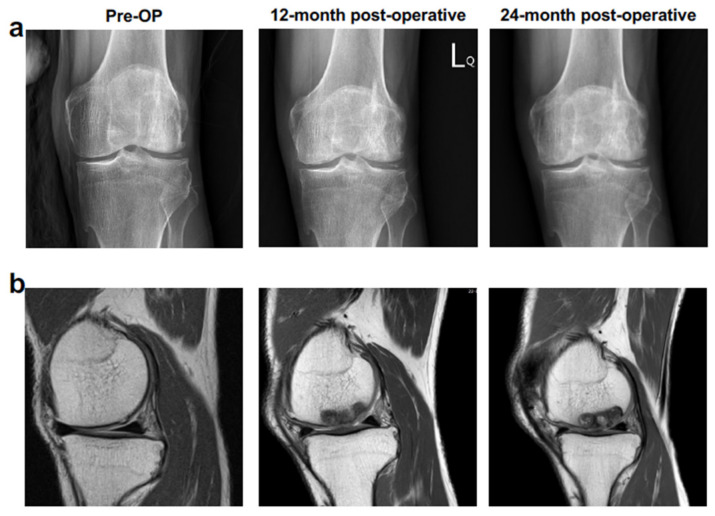
Representative radiological and MRI outcomes from a single illustrative patient (Patient 1, Medial Femoral Condyle lesion, 9 cm^2^, KL grade 2) at 24 months postoperatively. All eight patients underwent imaging evaluation; individual variation in bony remodeling and cartilage regeneration is reflected in Table 2 and Figure 6. (**a**) Plain roentgenography demonstrated radiopaque trabeculae within the original bony pit, consistent with ongoing bone remodeling. Complete bony bridging was not observed at this time point, consistent with the expected degradation timeline of the PLGA/β-TCP scaffold. (**b**) T1-weighted coronal MRI view demonstrating that the signal intensity within the bony pit remained distinct from adjacent native bone, consistent with incomplete scaffold resorption and ongoing subchondral remodeling.

All eight patients underwent radiography and MRI before surgery and at 12 and 24 months after surgery. Despite incomplete bony bridging, plain roentgenography demonstrated the presence of radiopaque trabeculae within the original bony pit at the grafted site, indicating ongoing bone remodeling (Figure 5a). The MRI signal of the content within the bony pit differed from that of the neighboring native bone, resulting in a blurred boundary that demarcated the bony graft site (Figure 5b).

**Figure 6:**
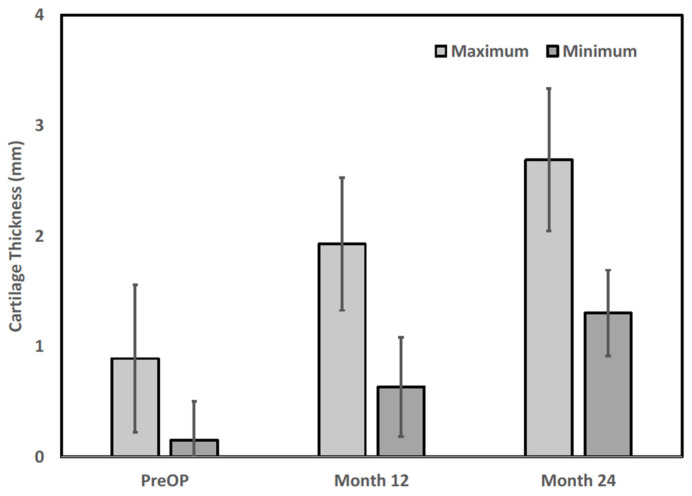
Maximal and minimal cartilage thickness at the repair site measured by the independent radiologist preoperatively and at 12 and 24 months postoperatively. Maximal thickness was measured at the thickest point of the repair tissue, and minimal thickness at the thinnest point within the repair area. Error bars represent mean ± standard deviation (*n* = 8).

Considerable inter-patient variability in cartilage thickness was observed. The preoperative maximal and minimal cartilage thicknesses were 0.9 ± 0.7 and 0.2 ± 0.4 mm, respectively, and gradually increased to 1.9 ± 0.6 and 0.6 ± 0.5 mm at 12 months postoperatively and to 2.7 ± 0.6 and 1.3 ± 0.4 mm at 24 months postoperatively (Figure 6; derived from quantitative MRI thickness mapping). Improvement at 12 and 24 months postoperatively was also observed in the MOCART subscale ‘filling of defect’, ‘integration to border’, ‘surface of repair tissue’, and ‘signal intensity of repair tissue’ (Table 2). Spearman correlation analysis between change in total MOCART score and change in IKDC score at 24 months revealed a positive association (r_s_ = 0.71, *p* = 0.048), suggesting that patients with greater radiological improvement in repair tissue quality also tended to report greater functional improvement. However, given the small cohort, this finding should be interpreted cautiously.

## 4. Discussion

The present study was designed as a single-center, open-label, single-arm feasibility trial to evaluate, for the first time, the use of RevoCart in an OA patient population. This study design was selected because the patient population (KL grade 1–3 OA) had not previously been included in RevoCart trials, necessitating a preliminary safety and feasibility evaluation before progressing to a controlled trial. The KOOS and IKDC scores were selected as primary outcome measures because they are validated, widely used patient-reported outcome tools for knee cartilage repair studies [25,26,27]. MOCART scoring was used as the standard radiological assessment for cartilage repair tissue quality [28].

The main objective of the present study was to evaluate the safety and effectiveness of RevoCart in patients with knee osteoarthritis. Minor adverse effects, including knee swelling and soreness, were documented six weeks postoperatively. However, these effects were resolved without any intervention at the third month of follow-up, consistent with the trials reported by Chiang et al. [21] and Tseng et al. [22], both of which utilized a biphasic construct with minced autologous cartilage that was similar to that in the present study. No recurring adverse effects or serious complications were recorded throughout the two-year follow-up period. This confirms the safety of the reparative surgery for patients with mild- to moderate-stage knee osteoarthritis.

To prepare the recipient holes for Chondroplug implantation, the cartilage was drilled to form pits at the lesion sites. It has been reported that surgically created bony lesions significantly weaken bone strength and may lead to an increased risk of pathologic fracture [29,30]. In the present study, however, there was no mechanical collapse of the load-bearing surface into the bone lesion. Therefore, it is essential to highlight that all patients’ bones at the surgical sites had enough strength despite the defect. Although the radiographic images at two years postoperatively revealed a lack of complete bony bridging at the surgical site, these images demonstrated that the bone had undergone some remodeling, resulting in the increase in bone density, formation of a bony bridge, and the improvement of bone strength. Another concern regarding Chondroplug is the potential hydrolysis of its major component, PLGA, which can create an acidic microenvironment, causing local inflammation that may be harmful to tissue regeneration and prognosis [31]. In the present study, however, such adverse effects were not observed, presumably owing to the extended degradation periods of PLGA and the neutralization of acidic degradation of PLGA by the ß-TCP [32], and, thus, the reduced accumulation of glycolic/lactic acid.

In the present study, mean IKDC scores and all mean KOOS subscales increased significantly at six months postoperatively, persisting throughout the residual follow-up period. Similarly, all mean VAS scores decreased considerably at six months postoperatively. These results demonstrate the preliminary clinical feasibility of RevoCart for treating knee OA patients with chondral or osteochondral lesions. The progression of improvements in KOOS and VAS scores in the patients in our study differed from that reported previously by Chiang et al. [21] and Tseng et al. [22], likely owing to the inclusion of an older patient population with greater degenerative change in the present trial. It should be noted that five of the eight patients underwent concomitant procedures (microdrilling in four patients and meniscal repair in one). Microdrilling, by stimulating the release of bone marrow-derived mesenchymal cells at the surgical site, may have independently contributed to cartilage repair and functional improvement. In the absence of a RevoCart-only control arm, the relative contributions of RevoCart and concomitant procedures to the observed outcomes cannot be separated. Future controlled studies should stratify patients by concomitant procedure type and minimize confounding by standardizing the surgical protocol.

Over the past few decades, there has been a proliferation of multi-phasic scaffolds designed to address osteochondral lesions [33,34,35,36]. Among the investigated scaffolds, the TruFit plug has been withdrawn from the market due to insufficient clinical improvement and poor graft integration [37]. In a meta-analysis of 18 studies (640 patients), Boffa et al. [38] reported mean improvements in IKDC score of 26.0 (95% CI: 23.3–28.8) and 31.1 (95% CI: 28.0–34.3) at 1- and 2-year follow-up for cell-free scaffolds (MaioRegen and Agili-C). In the present study, the mean improvements in IKDC score were 42.0 (95% CI: 32.1–51.9) and 39.4 (95% CI: 31.4–47.8) at 1- and 2-year follow-up, respectively, tentatively suggesting a potentially enhanced treatment effect associated with the incorporation of autologous minced cartilage tissue in the biphasic scaffold. The RevoCart system offers several notable features relative to existing approaches. It is a single-step intraoperative procedure, avoiding the two-stage operations required for MACI, thereby reducing costs and patient burden. The biphasic construct architecture addresses both the cartilage and subchondral bone simultaneously, which is particularly relevant in OA where subchondral pathology commonly accompanies cartilage loss. The biomechanical design of the construct, including its structural stability and load-bearing characteristics, is an important consideration in weight-bearing osteochondral applications; optimizing construct configuration for load distribution remains an area for future study [39]. Regarding biocompatibility, the PLGA/β-TCP scaffold composition has a well-established safety profile, and the absence of implant-related adverse biological responses in the present cohort is consistent with prior reports of favorable tissue integration with resorbable orthopedic biomaterials [40].

The radiological and imaging findings at two years indicated ongoing but incomplete osseous and cartilaginous remodeling at the graft site. Incomplete bony bridging and persistent inhomogeneous repair structure (MOCART “Structure” subscale) were observed in all patients throughout follow-up. Incomplete bony bridging at the scaffold–bone interface at 24 months is consistent with the known degradation timeline of the PLGA/β-TCP osseous phase, which undergoes gradual resorption over 12–24 months and is progressively replaced by native bone. The MRI signal heterogeneity at the bony pit likely reflects this ongoing remodeling process rather than implant failure, as no mechanical collapse was observed.

Persistent subchondral abnormalities and inhomogeneous repair tissue structure suggest that cartilage and bone remodeling are ongoing at 2 years, consistent with previous reports of multi-year maturation timelines for biphasic osteochondral constructs [22], and longer follow-up is required to determine whether the tissue structure will progressively normalize. Furthermore, a positive Spearman correlation between MOCART score change and IKDC score change at 24 months (r_s_ = 0.71, *p* = 0.048) suggests that radiological tissue quality may be a relevant predictor of functional outcome, though this relationship requires confirmation in larger studies.

This feasibility study is subject to several important limitations. First, the small sample size (*n* = 8) is the primary limitation, and represents the most significant constraint on the interpretability of these findings. The absence of a control group means that the observed improvements cannot be attributed exclusively to the RevoCart intervention, as contributions from placebo effects, postoperative rehabilitation, and the natural history of symptoms cannot be excluded. The single-center, open-label design and consecutive patient recruitment introduce selection bias and limit external validity. These results should therefore be regarded as preliminary observations that warrant confirmation in randomized controlled trials with larger, more diverse patient cohorts. Second, the evaluation of regenerated cartilage was based on MRI studies. MRI cannot reliably distinguish hyaline cartilage from fibrocartilage repair tissue, and histological analysis, which was not performed due to ethical concerns regarding biopsy, remains the definitive method for characterizing repair tissue quality. Nevertheless, the hyaline-like cartilage regeneration capability of RevoCart has been previously established by histological evidence [21,22,41]. These results should therefore be regarded as preliminary observations that warrant confirmation in larger, more diverse patient cohorts, with longer follow-up to evaluate the durability of cartilage regeneration in the context of mild-to-moderate osteoarthritis. Third, longer follow-up is warranted to assess the long-term outcomes of RevoCart treatment and the incidence of secondary osteoarthritis in these patients. Finally, the number of constructs used (1–3) varied across patients according to lesion size; the potential influence of construct number on outcome could not be assessed in this small cohort and should be evaluated in future studies. Future studies should also consider implementing standardized data management and implant traceability systems, such as blockchain-based platforms, which have been proposed as emerging tools to improve data integrity, procedural standardization, and long-term follow-up management in advanced orthopedic regenerative procedures [42].

## 5. Conclusions

In conclusion, this small-scale feasibility study provides preliminary evidence that RevoCart is clinically tolerable and associated with improvements in knee pain, function, and radiological cartilage repair at two years in patients with mild-to-moderate-stage knee osteoarthritis. These encouraging preliminary findings support the rationale for conducting larger, randomized controlled trials to further evaluate the clinical role of biphasic osteochondral constructs in this patient population.

## Figures and Tables

**Table 1:** Demographic data of study patients who received RevoCart.

Age	Gender	BMI	Lesion Location ^a^	Lesion Size (cm^2^)	ICRS Grade ^b^	KL Grade ^c^	Number of Implant	Conjoined Procedure
52.6	M	22.3	MFC	9	3	2	3	Microdrilling
55.9	M	31.2	LFC	9	3	2	2	Microdrilling
54.9	M	29.3	MFC	3	3	3	2	Microdrilling
62	F	27.5	MFC	3	3	3	2	Meniscus Repair
47.6	M	37.7	MFC	3.9	4	3	3	N/A
59.3	M	26	MFC	4	4	3	2	N/A
34.7	F	26	LFC	2	4	1	1	N/A
63.9	F	20.7	MFC	2	4	1	1	N/A

^a^ MFC: Medial Femoral Condyle; LFC: Lateral Femoral Condyle; ^b^ ICRS, International Cartilage Repair Society Score; ^c^ KL, Kellgren–Lawrence.

**Table 2:** MRI evaluation of the repaired lesion sites by MOCART scale at preoperation and at 12 and 24 months post operation.

Parameters	Number of Patients		
	Pre-Op	12 Months	24 Months
1. Filling of the defect			
Complete	0	0	0
Hypertrophy	0	4	5
Incomplete, >50%	0	4	3
Incomplete, <50%	3	0	0
Subchondral bone exposed	5	0	0
2. Integration to border			
Complete	0	0	1
Incomplete with split-like border	0	6	5
Incomplete with visible defect < 50%	1	2	2
Incomplete with visible defect > 50%	7	0	0
3. Surface of repair tissue			
Intact	0	1	2
Damaged < 50% of repair tissue depth	1	7	5
Damaged > 50% of repair tissue depth/total degeneration	7	0	0
4. Structure			
Homogeneous	0	0	0
Inhomogeneous or cleft formation	8	8	8
5. Signal intensity of repair tissue (dual T2-FSE)			
Isointense	0	2	1
Moderately hyperintense	3	6	6
Markedly hyperintense	5	0	1
6. Signal intensity of repair tissue (3D-GE-FS)			
Isointense	0	1	0
Moderately hyperintense	4	7	7
Markedly hyperintense	4	0	1
7. Subchondral lamina			
Intact	1	1	2
Not intact	7	7	6
8. Subchondral bone			
Intact	0	0	2
Not intact	8	8	6
9. Adhesion			
No	8	8	7
Yes	0	0	1
10. Effusion			
No	1	2	4
Yes	7	6	4

## Data Availability

Data are available upon reasonable request from the corresponding author due to patient privacy and ethical restrictions.
